# Modification of the mycobacteriophage Ms6 *attP *core allows the integration of multiple vectors into different tRNA^ala ^T-loops in slow- and fast-growing mycobacteria

**DOI:** 10.1186/1471-2199-7-47

**Published:** 2006-12-15

**Authors:** Tiago Dos Vultos, Isabelle Méderlé, Valérie Abadie, Madalena Pimentel, José Moniz-Pereira, Brigitte Gicquel, Jean-Marc Reyrat, Nathalie Winter

**Affiliations:** 1 Unité de Génétique Mycobactérienne, Institut Pasteur, 25, rue du Dr Roux, 75724 Paris Cedex 15, France; 2 Unidade dos Retrovirus e Infecções Associadas, Centro de Patogénese Molecular, Faculdade de Farmácia da Universidade de Lisboa, Lisbon, Portugal; 3 Inserm-U 570, Unité de Pathogénie des Infections Systémiques, Groupe Avenir, Université Paris V-Descartes, Faculté de Médecine René Descartes, Paris Cedex 15, F-75730, France

## Abstract

**Background:**

Mycobacteriophage Ms6 integrates into *Mycobacterium smegmatis *and *M. bovis *BCG chromosome at the 3' end of tRNA^ala ^genes. Homologous recombination occurs between the phage *attP *core and the *attB *site located in the T-loop. Integration-proficient vectors derived from Ms6 are useful genetic tools, but their insertion sites in the BCG chromosome remain poorly defined. The primary objective of this study was to identify Ms6 target genes in *M. smegmatis *and BCG. We then aimed to modify the *attP *site in Ms6-derived vectors, to switch integration to other tRNA^ala ^loci. This provided the basis for the development of recombinant *M. bovis *BCG strains expressing several reporter genes inserted into different tRNA^ala ^genes.

**Results:**

The three tRNA^ala ^genes are highly conserved in *M. smegmatis *and BCG. However, in the T-loop of tRNA^alaU ^and tRNA^alaV ^containing the *attB *site, a single base difference was observed between the two species. We observed that the tRNA^alaU ^gene was the only site into which Ms6-derived integration-proficient vectors integrated in *M. smegmatis*, whereas in BCG, the tRNA^alaV ^gene was used as the target. No integration occurred in the BCG tRNA^alaU ^T-loop, despite a difference of only one base from the 26-base Ms6 *attP *core. We mutated the *attP *core to give a perfect match with the other tRNA^ala ^T-loops from *M. smegmatis *and BCG. Modification of the seven-base T-loop decreased integration efficiency, identifying this site as a possible site of strand exchange. Finally, two Ms6 vectors were constructed to integrate two reporter genes into the tRNA^alaU ^and tRNA^alaV ^T-loops of the same BCG chromosome.

**Conclusion:**

Small changes in the 7 bp T-loop *attP *site of Ms6 made it possible to use another *attB *site, albeit with a lower integration efficiency. These molecular studies on BCG tRNA^ala ^genes made it possible to create valuable tools for the site-directed insertion of several genes in the same BCG strain. These tools will be useful for the development of novel multivalent vaccines and genetic studies.

## Background

Temperate phages integrate into the bacterial chromosome through a site-specific recombination event catalyzed by a phage-encoded recombinase. This process involves a common core present in the phage *attP *and the bacterial *attB *genomic DNA sequences, which are identical [1]. Genetic tools based on phage systems have furthered research into the biology of *Mycobacterium tuberculosis*, a pathogen responsible for about two million deaths each year [2]. L5 [3] and Ms6 [4], both temperate mycobacteriophages, integrate into genes encoding tRNAs. L5 integrates into a tRNA^gly ^gene in the genome of the fast-growing species *M. smegmatis *or the slow-growing species *M. bovis *Bacillus Calmette Guérin (BCG), which is used as a vaccine against tuberculosis [5,6]. Integration-proficient vectors containing the *attP *site and either a tyrosine-integrase [5,7] or a serine-integrase [8] system integrate into the *attB *site of the mycobacterial chromosome. Most of these integrative vectors do not retain recombination directionality factors from the phage that mediate excision [9] and are generally stably maintained in the mycobacterial genome, even in the absence of antibiotic selection [10]. However, plasmid loss can occur through low level integrase-mediated excision [11]. These vectors are of considerable use both for the development of recombinant BCG (rBCG) strains with long-term heterologous gene expression for vaccine development [10] and for overcoming the problems associated with the use of multicopy extrachromosomal plasmids in genetic studies. Ms6-derived vectors integrate efficiently into the genomes of both slow- and fast-growing mycobacteria. In *M. smegmatis*, the *attB *core site overlaps the 3' end of a tRNA^ala ^gene containing the 7 bp T-loop [7]. The precise point of strand exchange between *attP *and *attB *is unknown. *In silico *scanning of the genomes of *M. smegmatis *and *M. tuberculosis *showed that there were three tRNA^ala ^genes containing 3' end *attB *sequences either identical or similar to the Ms6 *attP *core site. We show here that Ms6 integrates into different tRNA^ala ^genes in *M. smegmatis *and BCG, according to the presence of identical *attB *and *attP *cores. We also used site-directed mutagenesis of the *attP *core to construct vectors integrating into tRNA^ala ^genes other than that targeted by the wild-type *attP*. This led to the development of a method for integrating two vectors, carrying different heterologous genes, into two different loci of the same BCG chromosome.

## Results

### Genetic organisation of tRNA^ala ^genes from *M. smegmatis *and BCG

A BLASTn search of the *M. tuberculosis *genome [12] for sequences similar to the Ms6 *attP *core region [13] revealed three possible *attB *sites at the 3' ends of three tRNA^ala ^genes, named after their anti codon: GGC for tRNA^alaU^, CGC for tRNA^alaV^, and TGC for tRNA^alaT^. In tRNA^alaV^, the *attB *core sequence was identical to the 26 bases of the *attP *core region [7]. In tRNA^alaU ^and tRNA^alaT^, one and three mismatches, respectively, were observed between the 26 bp Ms6 *attP *and *attB *core sequences. Alignment of the *attP *sequence with the genome of *M. bovis *[14] revealed the presence of the same three *attB *sites (data not shown). None of the tRNA^ala^genes mapped to deletion regions described in BCG [15,16].

Alignment of the Ms6 *attP *core sequence with the genome of *M. smegmatis *[17] revealed three possible *attB *sites displaying sequence similarities. As for BCG, the three possible *attB *sites were located in tRNA^alaU ^(anticodon GGC), tRNA^alaV ^(anticodon CGC) and tRNA^alaT ^(anticodon TGC). The three *M. smegmatis *and BCG tRNA^ala ^structures were analysed with tRNA-scan SE [18]. A sequence identity block common to *attP*-*attB *was identified that encompassed the tRNA^ala ^T-loop located at the 3' end, without extending to the variable region (see additional file 2).

**Figure 1:**
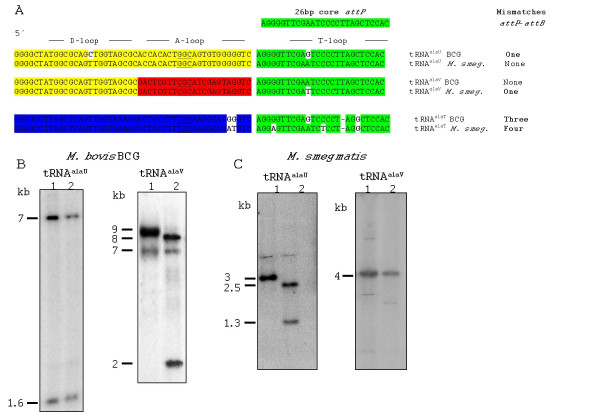
**Southern blot of genomic DNA from wild-type and pAV6950-transfected *M. bovis *BCG and *M. smegmatis *integrants**. **A**: Alignment between the three tRNA^ala ^gene sequences from *M. bovis *BCG or *M. smegmatis *and the Ms6 *att*P 26-base core sequence. The coloured blocks represent identical sequence stretches, base differences are indicated in bold and the anticodon is underlined. The position of anticodon and T-loops in the tRNA^ala ^sequence is indicated. **B**: Genomic DNA from wild-type BCG (lane 1) and a pAV-SIV integrant (lane 2) was probed with a PCR product based on the tRNA^alaU ^(left panel) or tRNA^alaV ^(right panel) sequence from the *M. tuberculosis *genome. **C**: Genomic DNA from wild-type *M. smegmatis *(lane 1) and a pAV-SIV integrant (lane 2) was probed with a PCR product based on the tRNA^alaU ^(left panel) or tRNA^alaV ^(right panel) sequence from the *M. smegmatis *genome.

In both species, the tRNA^ala^-encoding genes mapped to distant regions of the chromosome. The three tRNA^ala ^genes of *M. smegmatis *and BCG were highly similar, but not identical. Interestingly, the tRNA^alaV ^T-loop from BCG was identical to the tRNA^alaU ^T-loop from *M. smegmatis*. In both species, the T-loop from two other tRNA^ala ^genes differed from the 26 bp core *attP *site from Ms6 by one to four bases (Fig. 1A)

### The integration of Ms6-derived vectors requires 100% identity of the 26 base cores of *attP *and *attB*

We have previously described the construction of pAV-SIV, an Ms6-derived integration-proficient vector, for the production of rBCG candidate vaccines genetically stable *in vivo *and expressing genes from the simian immunodeficiency virus SIVmac251 [10]. Here, we analysed the insertion locus of pAV-SIV in the BCG chromosome. We carried out Southern blot analysis on genomic DNA using a probe (see additional file 1 for all primers plasmids and strains used in this study) designed from the tRNA^alaU ^gene from *M. tuberculosis*, as we previously showed that the target insertion sequence corresponded to tRNA^alaU ^[7]. However, we did observe no band disruption when the genomic DNA hybridisation profiles of pAV-SIV integrants and wild-type BCG were compared, indicating that the integration-proficient plasmids had not inserted into the tRNA^alaU ^gene (Fig. 1B, left panel). We then amplified another probe derived from the tRNA^alaV ^sequence and observed, on a Southern blot of DNA from wild-type BCG, two specific bands : a 9 kb band showing a strong positive signal and a weakly hybridizing 7 kb band (Fig. 1B, right panel). Following transformation with pAV-SIV, the 9 kb band disappeared, giving rise to two bands of 8 and 2 kb. The 7 kb band, due to cross-hybridisation of tRNA^alaU ^*attB *with the tRNA^alaV ^probe, remained intact. This confirmed the specific integration of pAV-SIV into the tRNA^alaV ^gene displaying identity with the *attP *rather than into the *attB *site from tRNA^alaU^, which has a base mismatch. We then investigated whether Ms6-derived vectors also targeted the *M. smegmatis attB *site displaying 100% identity with the Ms6 *attP *core – the tRNA^alaU ^gene. A Southern blot analysis with two probes containing either the tRNA^alaU ^or the tRNA^alaV ^gene sequence revealed that the 3 kb band hybridising with the tRNA^alaU ^probe in genomic DNA from wild-type *M. smegmatis *was disrupted in DNA from pAV-SIV integrants, giving 2.5 kb and 1.3 kb bands (Fig. 1C, left panel). When DNA was probed with tRNA^alaV^, no difference in hybridisation profile was observed between the wild type and integrants, demonstrating an absence of integration into the tRNA^alaV ^locus (Fig. 1C, right panel).

We investigated whether integration into an *attB *site carrying mismatches with the *attP *core site was possible, as reported for L5 [5], by designing three primer pairs to amplify tRNA^alaU^, tRNA^alaV ^or tRNA^alaT ^regions both from *M. smegmatis *and BCG genome sequences. The PCR amplification of genomic DNA from fifty pAV-SIV BCG integrants identified tRNA^alaV ^as the only gene target for Ms6 integration. By contrast, in the 50 *M. smegmatis *integrants analysed, pAV-SIV had inserted into the tRNA^alaU ^gene (data not shown).

### Modification of the *attP *core makes integration into the T-loop of other tRNA^ala ^genes possible

The tRNA^alaU ^gene (anticodon GGC) of BCG and the tRNA^alaV ^gene of *M. smegmatis *(anticodon CGC) differ from the *attP *core by one base in the T-loop, whereas the tRNA^alaT ^genes differ from this core sequence by three and four bases, respectively (Fig. 1A). We then investigated whether modification of the *attP *site to match these T-loops would allow us to target Ms6-derived integrative vectors to other tRNA^ala ^genes. Site-directed mutagenesis was performed on pAV6950, carrying the natural Ms6 *attP *core sequence, to construct four vectors carrying modified 26 bp *attP *cores identical to the various tRNA^ala ^T-loops.

In *M. smegmatis*, the integration efficiency of pSV, carrying an *attP *core identical to the tRNA^alaV ^T-loop, was only 12% that of the non-mutated pAV6950 targeting tRNA^alaU ^(Table 1). PCR amplification of *M. smegmatis *pSV integrants confirmed that the vector had targeted tRNA^alaV ^in all clones, as expected. When four base changes were introduced, to match the tRNA^alaT ^T-loop sequence (plasmid pST), integration efficiency was only 0.05% that with the non-mutated pAV6950.

In BCG, pBU, in which the sequence TTCGAA was mutated to TTCGAG to match the tRNA^alaU ^T-loop, integration efficiency was 34% that for the non-mutated pAV6950 targeting tRNA^alaV ^(Table 1). In all integrants, pBU targeted the tRNA^alaU ^T-loop, as expected. When three base changes were introduced, to match the tRNA^alaT ^T-loop, integration efficiency was 18% with respect to the non-mutated *attP*. In all clones tested, integration into tRNA^alaT ^had occurred.

### Ms6-derived vectors targeting two different tRNA^ala ^T-loops in BCG can be used to construct bivalent recombinant BCG strains

**Figure 2:**
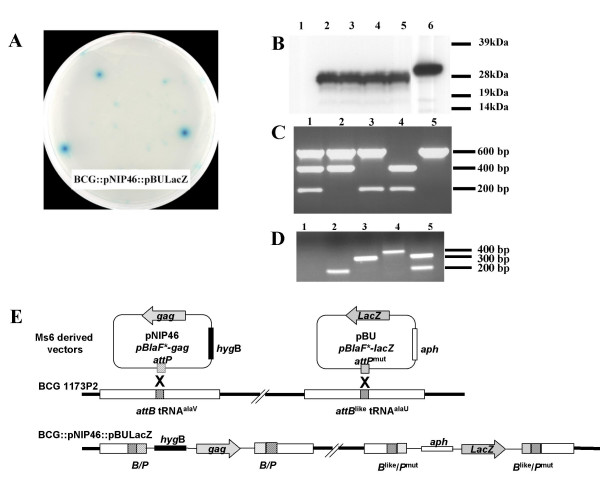
**Bivalent recombinant BCG strain containing two pAV6950-derived vectors inserted into the tRNA^alaU ^and tRNA^alaV ^T-loops**. BCG was transformed with the two vectors – pNIP46, targeting tRNA^alaV^, and pBU-*lacZ*, targeting tRNA^alaU ^– to obtain a strain coexpressing the two foreign genes. **A**: Expression of the *lacZ *gene in rBCG::pNIP46::pBU-*lacZ *was detected as blue coloration on agar plates supplemented with the β-galactosidase substrate X-gal. **B**: Expression of *gag*p26 was detected by western blotting and wild-type BCG (lane 1), rBCG::pNIP46, (lane 2) and three different clones of rBCG::pNIP46::pBU-*lacZ *(lanes 3, 4, 5) were analysed. Lane 6 corresponds to purified recombinant Gagp26 produced in *E. coli *with an N-terminal His-tag. **C**: Multiplex PCR using the three sets of primers located upstream and downstream from the three different *attB *sites in BCG. Absence of the 200 bp, 400 bp or 600 bp amplification products indicates disruption of the tRNA^alaU^, tRNA^alaV ^and tRNA^alaT ^T-loops, respectively. The three fragments were present in the wild-type BCG chromosome (lane 1). After a single integration of pBU-*lacZ *into tRNA^alaU^, only the 600 and 400 bp fragments were present (lane 2), and in BCG::pNIP46, in which tRNA^alaV ^had been disrupted, both the 600 and 200 bp fragments were amplified (lane 3). pBT disrupted the tRNA^alaT ^locus (lane 4). In the double integrant, BCG::pNIP46::pBU-*lacZ*, only the tRNA^alaT ^locus was intact and gave the 600 bp amplification fragment (lane 5). **D **Integration of each vector was confirmed by PCR using a mixture of the three primers upstream from tRNA^alaU^, tRNA^alaV ^and tRNA^alaT ^combined with LM2, annealing to the Ms6 integrase. No amplification was detected in wild type BCG chromosome (lane 1), by contrast, single products were detected in BCG::pBU-*lacZ *(lane 2), BCG::pNIP46 (lane 3) and BCG::pBT (lane 4). In BCG::pNIP46::pBU-*lacZ *two bands were obtained confirming the double integration (lane 5). **E **A diagram of the double integration of pNIP46 and pBU-*lacZ *is provided

As pBU made possible integration into tRNA^alaU^, we investigated whether both tRNA^alaU ^and tRNA^alaV ^could be targeted in the same BCG strain. BCG was electroporated with pNIP46, a pAV6950-derived vector (targeting the tRNA^alaV ^T-loop in BCG) containing the SIVmac251 *gag*p26 gene and a gene conferring resistance to hygromycin. The recombinant BCG::pNIP46 strain expressing *gag*p26 was then transformed with pBU-*lacZ*, containing the *Escherichia coli lacZ *and a kanamycin resistance gene. Recombinant clones, selected on medium supplemented with kanamycin and X-gal, displayed β-galactosidase activity (Fig. 2A). The expression of *gag*p26 was also detected in these clones by western blotting (Fig. 2B). PCR was used to determine the site of integration of the various plasmids in the BCG integrants (Fig. 2C). It was found that pNIP46 had inserted into tRNA^alaV ^and pBU-*lacZ *into tRNA^alaU^. In the double integrant, BCG::pNIP46::pBU-*lacZ*, both tRNA^alaU ^and tRNA^alaV ^were disrupted. A diagram of this double integration of an Ms6-derived vector carrying natural *attP *in BCG tRNA^alaV ^and an Ms6-derived vector with modified *attP *in BCG tRNA^alaU ^is provided in Figure 2D.

## Discussion

Determination of the complete genome sequences of *M. tuberculosis *[12] and *M. smegmatis *[17] made it possibe to analyse the possible integration targets of Ms6-derived vectors. In contrast to expectations [7], we observed that Ms6 targeted two different tRNA^ala ^genes in the fast-growing species *M. smegmatis *and the slow-growing species *M. bovis *BCG. The critical factor defining the exclusive integration site was the presence in the *attB *region of a core sequence identical to that of Ms6 *attP*. Thus, whereas in *M. smegmatis *100% of the transformants obtained with Ms6-derived integration-proficient vectors displayed insertion into tRNA^alaU^, in BCG, all transformants carried the vector in tRNA^alaV^. In the case of L5, another temperate mycobacteriophage widely used for genetic studies [3], integration into the *attB *site of the BCG chromosome may occur in a tRNA^glyV ^gene carrying one mismatch with the *attP *minimal core [5]. However, analysis of the three tRNA^gly ^genes of BCG showed that there was no tRNA^gly ^carrying an *attB *sequence identical to L5 *attP *in BCG (data not shown). L5-derived vectors therefore target an *attB *sequence carrying one mismatch with *attP*. This mismatch does not seem to affect the efficiency of L5 integration in BCG [5]. However, this mismatch maps outside the tRNA^glyV ^7 bp anticodon loop in which strand exchange beween *attP *and *attB *occurs [6].

The three tRNA^ala ^gene sequences displayed a high degree of similarity between *M. smegmatis *and BCG. Only two base differences in tRNA^alaU ^and one base difference in tRNA^alaV ^were observed. Interestingly, whereas the first 25 bases of the tRNA^alaU ^and tRNA^alaV ^anticodon loops were identical, the nucleotides involved in the formation of anticodon loop flanking symmetry differed in the tRNA^alaU ^and tRNA^alaV ^genes. This may be a signature of ancestral recombination between the tRNA^ala ^genes during evolution. The T-loops were remarkably conserved between tRNA^alaU ^and tRNA^alaV ^and between the species. However, one base difference occurred in this region containing the *attB *site. Point mutations occurring separately in the two species during evolution may account for these observations. Alternatively, the Ms6 phage or its ancestors may have introduced some of these mutations, as mycobacteriophages have actively participated in remodelling of the bacterial chromosome [19].

We next investigated whether the two "*attB*-like" sequences located in the two other tRNA^ala ^T-loops were potential target sites for modified Ms6. Indeed, with an integrase trained on one gene, a small change in the *attP *sequence may allow the element to switch to another tRNA^ala ^gene. We therefore mutated the 26-base *attP *core and assessed integration into the other mycobacterial loci. In BCG, one base difference between natural *attP *(TTCGAA) and pBU *attP*-like (TTCGAG) sequences reduced integration efficiency by 70%, despite identity with *attB *(tRNA^alaU^). The mutation introduced in *attP *was located in the 7 bp identical to the T-loop of the tRNA^ala ^gene but did not directly affect flanking symmetry (for review [1]. This change, although minor in nature, caused a dramatic drop in integration efficiency. Two additional changes in the 26-base sequence (pBT) only slightly reduced integration efficiency further in BCG, from the 70% decrease with pBU (one base change) to an 80% decrease with pBT (three base changes). This identifies the 7 bp T-loop sequence TTCGAA as important for Ms6 integration. Indeed, when *M. smegmatis *was electroporated with pBU (TTCGAG), despite there being only one base difference between this mutated *attP *and the three *attB *sites available in the genome, very few integrants were obtained (99% decrease in efficiency). Phages have been classified according to integration site sublocation within tRNA or tmRNA genes [20]. Class I phage integrase targets the tRNA anticodon loop whereas class II targets the tRNA T-loop. The precise crossover segment has been examined in class I phages, in which it maps to the 7 bp anticodon-encoding loop. This is also the case for mycobacteriophage L5 [6], which belongs to class I [20]. The precise location for crossover in class II phages remains unknown. Our data suggest that strand exchange between the class II phage Ms6 *attP *and mycobacterial *attB *occurs precisely in the 7 bp T-loop from tRNA^ala^.

Integration-proficient vectors derived from the temperate mycobacteriophage Ms6 are particularly useful for constructing recombinant BCG strains that are genetically stable *in vivo *[10]. BCG is also of considerable interest for use in the development of vaccines for simultaneous immunisation against several pathogens. The next step was to use Ms6 integration-proficient vectors targeting different tRNA^ala ^T-loops in BCG to construct multivalent vaccine strains. We therefore constructed one vector carrying the natural *attP *core (TTCGAA/tRNA^alaV^), a SIVmac251 gene encoding Gagp26 and another carrying an *attP *site targeting tRNA^alaU ^(TTCGAG) and carrying the *lacZ *gene from *E. coli*. In the rBCG::pNIP46::pBU-*lacZ *strain transformed with the two vectors, we observed expression of both the *gag*p26 and *lacZ *genes. Interestingly, the level of expression of *gag*p26 in the bivalent strain was similar to that observed in the monovalent rBCG::pNIP46 strain. This method may therefore be useful for the future development of new rBCG vaccines carrying multiple heterologous genes that will help in immunisation programmes.

## Methods

### Bacterial strains and plasmids

The *E. coli*, *M. smegmatis*, *M. bovis *BCG strains and the plasmids and primers used in this study are described in additional file 1. Mycobacterial strains were electroporated as previously described [21]. The plasmid pAV-SIV [10] was derived from pAV6950 [7], containing the *attP*-*int *region from mycobacteriophage Ms6 and an origin of replication from *E. coli*. It contains SIVmac251 genes inserted into the single *EcoR*I site of pAV6950 located upstream from the *attP *core and *int *gene. Similarly, pBU-*lacZ *contains the gene encoding β-galactosidase from *E. coli *inserted upstream from the modified *attP *core. Both pAV-SIV and pBU-*lacZ *express the *aph*3' gene from *Tn*5, conferring resistance to kanamycin (20 μg ml^-1^). pNIP46 is a pAV6950 derivative containing the *hyg*B gene from *Streptomyces hygrospicus *conferring resistance to hygromycin (50 μg ml^-1^) and the *gag*p26 gene from SIVmac251. BCG transformants were selected on solidified Middlebrook 7H11 medium (Difco) supplemented with OADC (0.05% oleic acid, 5% bovine serum albumin fraction V, 2% dextrose, 0.004% beef catalase, 0.85% NaCl) and the appropriate antibiotic. β-galactosidase activity in BCG transformed with pBU-lacZ was visualised by adding X-gal (80 μg ml^-1^, MP Biomedicals, Inc.) to the medium.

### PCR, Southern blotting and site-directed mutagenesis

PCR was performed in a final volume of 50 μl containing chromosomal DNA, 1.5 mM MgCl_2_, 200 μM of each dNTP, 50 pmoles of each primer and 1 unit of polymerase. Thirty-five cycles of [94°C 30 seconds, 60°C 30 seconds, 72°C 90 seconds] were run. For Southern blot analysis, genomic DNA from BCG and *M. smegmatis *pAV-SIV integrants was digested with *Bam*HI for 18 hours at 37°C. Probes were generated by PCR amplification (see primers in Table 1), using *M. tuberculosis *cosmid 22D7 for tRNA^alaU ^or cosmid 237 for tRNA^alaV ^or genomic *M. smegmatis *DNA. For site-directed mutagenesis of the *attP *core site, the QuickChange™ Site-Directed Mutagenesis kit from Stratagene (La Jolla, CA) was used according to the manufacturer's instructions. This procedure uses double-stranded DNA and two primers complementary to opposite strands of the vector and each containing the desired mutation. For site-directed mutagenesis, only the sequence primers identical to the *attP *core site are listed in additional file 1. Both this primer and the complementary primer were used in the reaction.

### Western blotting

Recombinant BCG colonies were grown at 37°C in Middlebrook 7H9 (Difco) medium supplemented with 10% ADC (5% bovine serum albumin fraction V, 2% dextrose, 0.003% beef catalase) and 0.05% Tween 80. We evaluated *gag*p26 expression with total protein extracts prepared as previously described [21]. Gagp26 was detected by incubation with a 1:500 dilution of polyclonal rabbit anti-Gag antibody followed by a 1:10000 dilution of anti-rabbit peroxidase-conjugated IgG (Amersham) and visualisation with an enhanced chemiluminescence kit (Amersham).

## Abbreviations

BCG: Bacillus Calmette-Guérin; rBCG: recombinant BCG; SIV: simian immunodeficiency virus.

## Authors' contributions

TDV and IM carried out the molecular and genetic studies and wrote the draft manuscript. They contributed equally to the work. VA carried out the southern blots. MP and JMP were involved in designing the studies. BG participated in the design of the study and contributed to the draft version of the manuscript. JMR participated in the design of the study, contributed to data analysis and helped with the writing of the draft manuscript. NW had the initial idea, co-ordinated the study and wrote the final version of the manuscript. All authors have read and approved the final manuscript.

## Supplementary Material

Additional File 1 Table S1. List of bacterial strains, primers and plasmids used in this study. Click here for file

Additional File 2 tRNA^ala ^secondary structures. Cartoon of the tRNA^ala ^genes secondary structures of *M. smegmatis *and *M. bovis *BCG designed by the tRNA-scan SE program [14]. Click here for file

## Figures and Tables

**Table 1:** Integration efficiency of vectors carrying *attP *core modifications

**Vector**	**26 bp core *attP***^a^	**Integration efficiency/pAV6950**^b^		**% integration into the tRNA**^**ala **^**T-loop**^c^	
		*M. smegmatis*	*M. bovis BCG*	*M. smegmatis*	*M. bovis *BCG
pAV6950	AGGGGTTCGAATCCCCTTAGCTCCAC	100%	100%	100% in tRNA^alaU^	100% in tRNA^alaV^
pBU	AGGGGTTCGA**G**TCCCCTTAGCTCCAC	1.1%	34%	ND	100% in alaU
pBT	AGGGGTTCGA**G**TCCCCT**AG**GCTCCAC	0.7%	18%	ND	100% in tRNA^alaT^
pSV	AGGGGTTCGA**T**TCCCCTTAGCTCCAC	12%	0.8%	100% tRNA^alaV^	ND
pST	AGG**A**GTTCGAATC**T**CCT**AG**GCTCCAC	0.05%	ND	ND	ND

**a: **Changes introduced by site-directed mutagenesis, with respect to the original *attP *site from pAV6950 are indicated in bold

**b**: Identical amounts of plasmid DNA were used to electroporate competent *M. smegmatis *or BCG. Integrants were numbered and the percentage of [integrants with modified *attP*/integrants with pAV6950] was calculated.

**c**: The insertion locus was determined by PCR amplification and the percentage of clones in the given tRNA^ala ^T-loop was calculated
