# Supplementary material for: Modification of the mycobacteriophage Ms6 attP core allows the integration of multiple vectors into different tRNAala T-loops in slow- and fast-growing mycobacteria
Source: BMC Mol Biol. 2006 Dec 15;7:47. doi: 10.1186/1471-2199-7-47 (PMC1762012; doi:10.1186/1471-2199-7-47)
Supplement: Additional File 1 — Table S1. List of bacterial strains, primers and plasmids used in this study. [file 1471-2199-7-47-S1.doc]

#### Table S1 : list of bacterial strains, primers and plasmids used in this study

| **Bacterial strains** |  | **Main characteristics** | **Source or reference** | |
| --- | --- | --- | --- | --- |
| *M. bovis* BCG |  | Pasteur strain; 1173P2 isolate | [1] | |
| *M. smegmatis* mc^2^155 |  | Mutant of *M. smegmatis* highly efficient for plasmid transformation | [2] | |
| *E.coli* XL1-Blue  rBCG ::pNIP46  rBCG ::pNIP46 ::pBU-*lacZ* |  | F'::Tn*10proA*^+^B^+^*lacIq*Δ(*lacZ*)*M15/recA1endA1gyrA96*(NalR)*thihsdR17*(rk-mk+)*supE44relA1lac*  Recombinant BCG strain harbouring pNIP46, resistant to hygromycin and expressing the SIVmac251*gag*p26 gene.  Recombinant BCG strain harbouring pNIP46 and pBU-*lacZ*, resistant to hygromycin and kanamycin, expressing the SIVmac251*gag*p26 and *E . coli lacZ* genes. | [3]  This study  This study | |
| **Plasmids** |  | **Main characteristics** | **Source or reference** | |
|  | pAV6950 | *E.coli* replicative/mycobacteria integrative shuttle vector. Contains the attachment site and the gene coding for integrase of mycobacteriophage Ms6. Carries the kanamycin resistance gene from Tn*5*. | [4] | |
|  | pAV-SIV | pAV6950 derivative. Contains SIVmac251 genes. | [5] | |
|  | pBU | pAV6950 derivative with mutated core sequence (see primer below) | This study | |
|  | pBT | pAV6950 derivative with mutated core sequence (see primer below) | This study | |
|  | pSV | pAV6950 derivative with mutated core sequence (see primer below) | This study | |
|  | pST | pAV6950 derivative with mutated core sequence (see primer below) | This study | |
|  | pBU-*lacZ* | pBU derivative containing the *lacZ* gene from *E. coli* cloned downstream from the *pBlaF** promoter of *M. fortuitum* | This study | |
|  | pNIP46 | pAV6950 derivative containing the hygromycin resistance gene from *Streptomyces hygrospicus* and the *gag*p26-encoding gene from SIVmac251 cloned downstream from *pBlaF** | This study | |
| **Primers used for:** | Name | **Sequence (5' to 3')** |  | |
| Site-directed mutagenesis | BU | AGGGGTTCGAGTCCCCTTAGCTCCAC |  |  |
| Site-directed mutagenesis | BT | AGGGGTTCGAGTCCCCTAGGCTCCAC |  |  |
| Site-directed mutagenesis | SV | AGGGGTTCGATTCCCCTTAGCTCCAC |  |  |
| Site-directed mutagenesis | ST | AGGAGTTCGAATCTCCTAGGCTCCAC |  |  |
| BCG tRNA^alaU^ probing | For | CGAGATCTACGACGCGGAGTCCTTCC |  | |
| BCG tRNA^alaU^ probing | Rev | CGGAATTCGTCTACAACGGCAAGATGG |  | |
| BCG tRNA^alaV^ probing | For | CGCAGTTCGGCCATCGGCG |  |  |
| BCG tRNA^alaV^ probing | Rev | GCCGGCGCCTCGACGTGG |  |
| *M. smegmatis* tRNA^alaU^ probing | For | AGGTGCCCTGACCAGCCATTTT |  |  |
| *M. smegmatis* tRNA^alaU^ probing | Rev | CTGACTCGAGGAAACGACGAAAC |  |
| *M. smegmatis* tRNA^alaV^ probing | For | TGGTGCTCGACATCGACGACGA |  | |
| *M. smegmatis* tRNA^alaV^ probing  BCG tRNA^alaU^ region amplification  BCG tRNA^alaU^ region amplification  BCG tRNA^alaV^ region amplification  BCG tRNA^alaV^ region amplification  BCG tRNA^alaT^ region amplification  BCG tRNA^alaT^ region amplification  Ms6 *int* gene region amplification | Rev  BalaUF  BalaUR  BalaVF  BalaVR  BalaTF  BalaTR  LM2 | GAGCAGTGCCTGTGAATAGTTG  GAGGCTCGCTGACCTGGGGC  CGTCTCGATTCGCACGTATTGTC  GGGCGCGGTGGTCCTGGAGA  ACTTTACCGGTACCACAAGGCC  CGGGCGCCGGTTGCGGTAAT  GACGCTACCGGGAAGCCGGC  GGCATCAGTGCGTGAACGGGA |  | |

# References for Table S1

1. Gheorghiu M, Augier J, Lagrange PH: **Maintenance and control of the French BCG strain 1173P2 (primary and secondary seed-lots).** *Bull Inst Past* 1983, **81**:281-288.

2. Snapper SB, Melton RE, Mustafa AS, Kieser T, Jacobs WR, Jr.: **Isolation and characterization of efficient plasmid transformation mutants of *Mycobacterium smegmatis***. *Mol Microbiol* 1990, **4**:1911-1919.

3. Bullock W, Fernandez J, Short J: **A high efficiency plasmid transforming recA *Escherichia coli* strain with beta-galactosidase selection**. *BioTechniques* 1987, **5**:376-380.

4. Freitas-Vieira A, Anes E, Moniz-Pereira J: **The site-specific recombination locus of mycobacteriophage Ms6 determines DNA integration at the tRNA(Ala) gene of *Mycobacterium* spp.** *Microbiology* 1998, **144 (Pt 12)**:3397-3406.

5. Méderlé I, Bourguin I, Ensergueix D, Badell E, Moniz-Pereira J, Gicquel B, Winter N: **Plasmidic versus insertional cloning of heterologous genes in *Mycobacterium bovis* BCG: impact on *in vivo* antigen persistence and immune responses.** *Infect Immun* 2002, **70**(1):303-314.
