# Supplementary material for: Modification of the mycobacteriophage Ms6 attP core allows the integration of multiple vectors into different tRNAala T-loops in slow- and fast-growing mycobacteria
Source: BMC Mol Biol. 2006 Dec 15;7:47. doi: 10.1186/1471-2199-7-47 (PMC1762012; doi:10.1186/1471-2199-7-47)
Supplement: Supplementary file 2 [file 1471-2199-7-47-S2.doc]

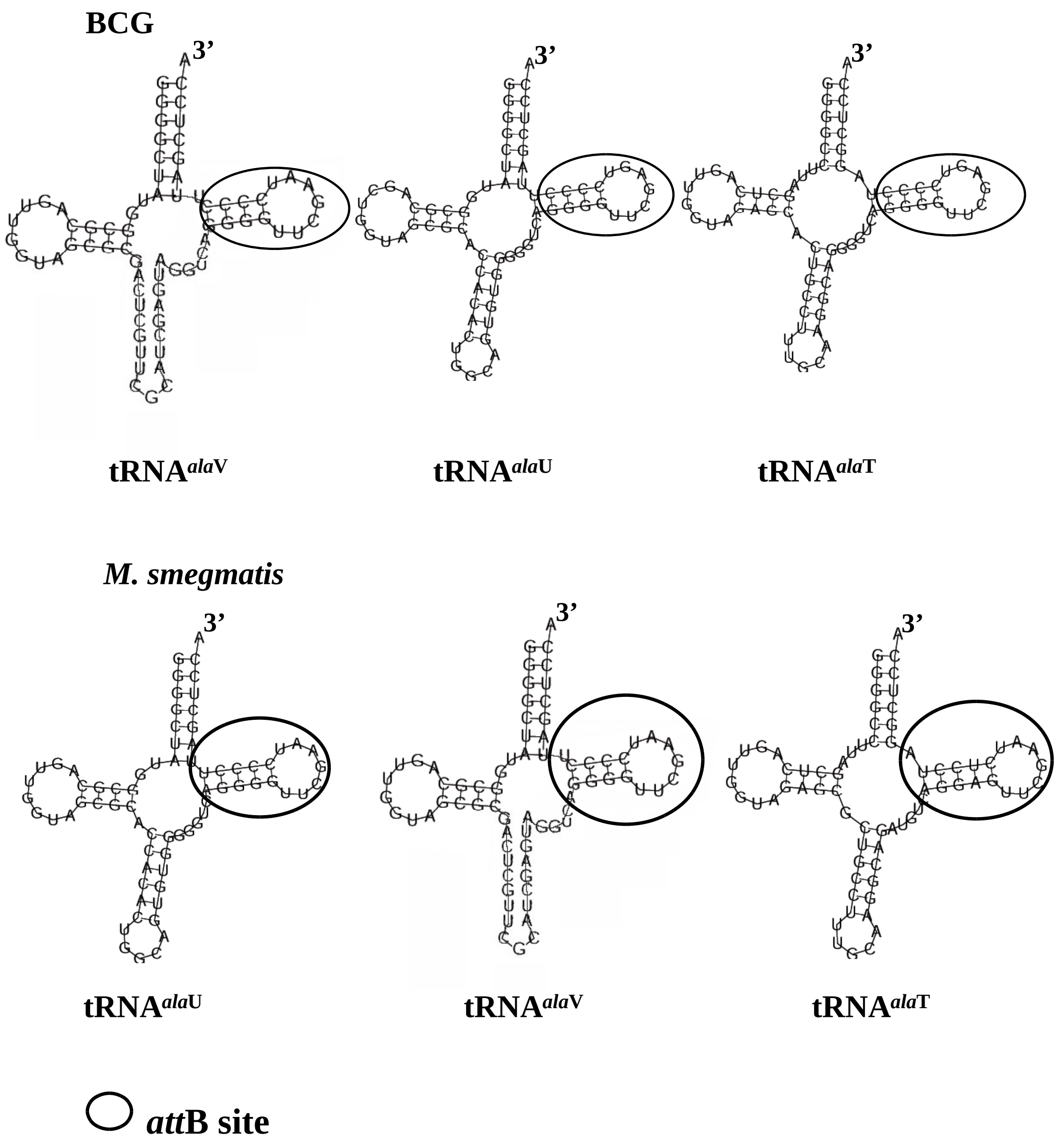
BCG
3'
tRNA*ala*V
tRNA*ala*U
tRNA*ala*T
M. smegmatis
tRNA*ala*U
tRNA*ala*V
tRNA*ala*T
*att*B site
